# Genome-wide association meta-analysis of functional outcome after ischemic stroke

**DOI:** 10.1212/WNL.0000000000007138

**Published:** 2019-03-19

**Authors:** Martin Söderholm, Annie Pedersen, Erik Lorentzen, Tara M. Stanne, Steve Bevan, Maja Olsson, John W. Cole, Israel Fernandez-Cadenas, Graeme J. Hankey, Jordi Jimenez-Conde, Katarina Jood, Jin-Moo Lee, Robin Lemmens, Christopher Levi, Braxton D. Mitchell, Bo Norrving, Kristiina Rannikmäe, Natalia S. Rost, Jonathan Rosand, Peter M. Rothwell, Rodney Scott, Daniel Strbian, Jonathan W. Sturm, Cathie Sudlow, Matthew Traylor, Vincent Thijs, Turgut Tatlisumak, Daniel Woo, Bradford B. Worrall, Jane M. Maguire, Arne Lindgren, Christina Jern

## Abstract

**Objective:**

To discover common genetic variants associated with poststroke outcomes using a genome-wide association (GWA) study.

**Methods:**

The study comprised 6,165 patients with ischemic stroke from 12 studies in Europe, the United States, and Australia included in the GISCOME (Genetics of Ischaemic Stroke Functional Outcome) network. The primary outcome was modified Rankin Scale score after 60 to 190 days, evaluated as 2 dichotomous variables (0–2 vs 3–6 and 0–1 vs 2–6) and subsequently as an ordinal variable. GWA analyses were performed in each study independently and results were meta-analyzed. Analyses were adjusted for age, sex, stroke severity (baseline NIH Stroke Scale score), and ancestry. The significance level was *p* < 5 × 10^−8^.

**Results:**

We identified one genetic variant associated with functional outcome with genome-wide significance (modified Rankin Scale scores 0–2 vs 3–6, *p* = 5.3 × 10^−9^). This intronic variant (rs1842681) in the *LOC105372028* gene is a previously reported trans-expression quantitative trait locus for *PPP1R21*, which encodes a regulatory subunit of protein phosphatase 1. This ubiquitous phosphatase is implicated in brain functions such as brain plasticity. Several variants detected in this study demonstrated suggestive association with outcome (*p* < 10^−5^), some of which are within or near genes with experimental evidence of influence on ischemic stroke volume and/or brain recovery (e.g., *NTN4*, *TEK*, and *PTCH1*).

**Conclusions:**

In this large GWA study on functional outcome after ischemic stroke, we report one significant variant and several variants with suggestive association to outcome 3 months after stroke onset with plausible mechanistic links to poststroke recovery. Future replication studies and exploration of potential functional mechanisms for identified genetic variants are warranted.


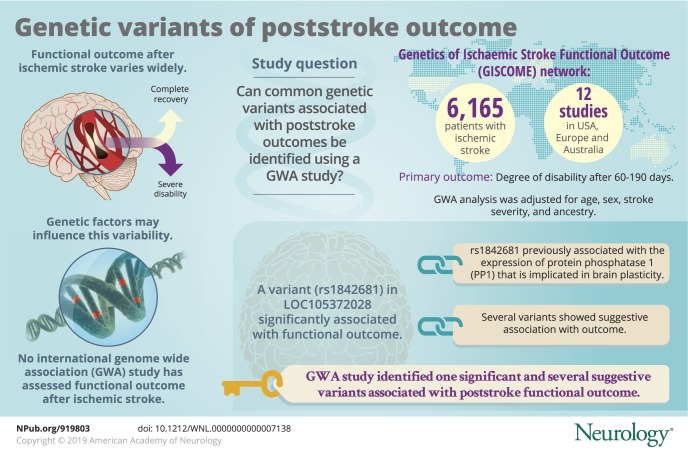


Functional outcomes after ischemic stroke have a wide range of interindividual variability, from complete recovery to persistent severe disability.^1^ Although a large number of factors influence recovery after ischemic stroke, such as age and initial stroke severity, a discernible portion of this interindividual variation remains unexplained by clinical factors.^1^ The unexplored mechanisms behind this variation represent potential keys in the search for a personalized poststroke management approach that would include accurate prognostic prediction as well as patient-tailored treatment, secondary prevention, and rehabilitation.

Genetic factors may account for part of the variability in stroke outcomes. Studies in humans and animals support a genetic influence on recovery after brain injury.^2–4^ In addition, genetic variants identified through candidate gene studies have been reported to associate with functional outcome after stroke, e.g., within the brain-derived neurotrophic factor and cyclooxygenase-2 genes,^5–7^ although these candidate gene study results have been inconsistent and need replication.^1^ To date, no genome-wide association (GWA) study has been published on functional outcome after overall ischemic stroke. Such a hypothesis-free approach may identify variants indicating novel pathways for poststroke pathophysiologic processes or recovery, and thereby suggest new targets for interventions and drug development.

The Genetics of Ischaemic Stroke Functional Outcome (GISCOME) network aims to identify genetic variants that may influence functional outcome after ischemic stroke.^8^ Here, we present the results from a GWA study of functional outcome as assessed by the modified Rankin Scale (mRS) at 3 months after ischemic stroke.

## Methods

### Study population

The GISCOME study population and analysis plan have been previously described in detail.^8^ In brief, the GISCOME study population consists of patients with ischemic stroke of mainly European ancestry aged 18 years and older from 12 study locations in Europe, the United States, and Australia. After the publication of the GISCOME study protocol,^8^ 2 additional sets of data from (1) the Sahlgrenska Academy Study on Ischemic Stroke, and (2) the Malmö Diet and Cancer Study have been added (data available from Dryad, supplemental Methods and tables e-1 and e-2, doi.org/10.5061/dryad.s38kf65). The GISCOME study includes centers with mRS and genotype data contributing to the International Stroke Genetics Consortium and the National Institute of Neurological Disorders and Stroke – Stroke Genetics Network (SiGN) study efforts studying genetics of stroke risk. We did not have any additional cohorts available at the time of this study for replication.

### Standard protocol approvals, registrations, and patient consents

All participants provided written informed consent to participate. For participants who were unable to communicate, consent was obtained from their next of kin. Local ethics committees approved the individual studies.

### Outcome

The mRS as close as possible to 90 days (60–190 days permitted) was selected to assess functional outcome, as described in the analysis plan.^8^ The majority of the included studies (≈80%) assessed mRS at 3 months ± 2 weeks.^8^ In most studies, this was done by face-to-face interviews. In 3 studies (Lund Stroke Register, Malmö Diet and Cancer Study, and parts of the Sahlgrenska Academy Study on Ischemic Stroke), data from the Swedish quality register for stroke (Riksstroke) were used to assess mRS by a validated translation algorithm.^8,9^ This approach prevented a differentiation between the mRS scores 0, 1, and 2.

Based on our a priori analysis plan, we analyzed mRS as 2 dichotomous variables, (1) mRS 0–2 vs 3–6 and (2) mRS 0–1 vs 2–6, and also as the full ordinal scale variable. The mRS 0–2 vs 3–6 and ordinal scale analyses included a larger number of participants. Analyses of mRS 0–1 vs 2–6 were performed to explore whether a second dichotomization could identify any strong associations that were not picked up by the other analyses. In addition, we made an explorative effort to investigate potential associations with infarcts in subcortical and cortical locations separately. Since information about infarct location was not available, we divided the cases into small vessel disease (referred to as lacunar stroke) and other subtypes (referred to as nonlacunar stroke), according to subtype classifications in TOAST (Trial of ORG 10172 in Acute Stroke Treatment).^10^ A total of 992 patients had lacunar stroke, 3,991 patients had nonlacunar stroke, and for 1,182 individuals, this information was missing.

### GWA analysis and meta-analysis

The methods for genotyping, imputation, and quality control of genotype data are described in data available from Dryad (supplemental Methods and tables e-1 and e-2, doi.org/10.5061/dryad.s38kf65). Multivariable models were used for analyses of each outcome variable, under an additive genetic model. We aimed to explore genetic variants associated with functional outcome that were independent of stroke severity. In this primary model, results were adjusted for age, sex, ancestry (up to the first 5 principal components [data available from Dryad, table e-3]), and baseline stroke severity as assessed by the NIH Stroke Scale (NIHSS) at 0 to 10 days post stroke onset, with preference to as close to day 0–1 as possible.^8^ In addition, models without adjustment for baseline stroke severity were performed for each outcome for comparison; however, unless otherwise stated, all results in this report refer to the primary model as described above.

All dichotomized analyses were performed with logistic regression using PLINK software version 1.90b4.6.^11^ The full mRS was analyzed with ordinal logistic regression under a cumulative logit model using the MATLAB mnrfit algorithm (MATLAB Statistics and Machine Learning Toolbox Release 2016b; The MathWorks, Inc., Natick, MA). Under the proportional odds assumption, the effect of a predictor single-nucleotide polymorphism (SNP) is invariant of the choice of outcome categories. As a result, estimates from all study cohorts, including those that did not separate mRS 0–2, are comparable in ordinal regression. Furthermore, one unit change in the dosage of a variant with regression coefficient β suggests a change in the odds of an mRS score lower than or equal to *x* vs higher than *x* by a factor 
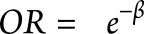
, equally for all scores *x*. We tested deviations from proportional odds for all reported variants from the ordinal models. No significant deviations were found.

**Figure 1:**
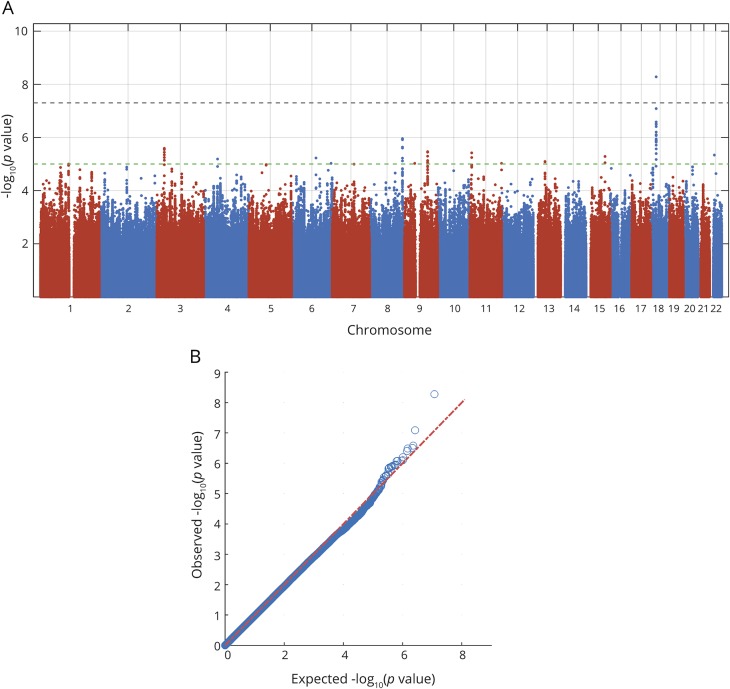
Manhattan and quantile-quantile plots of analysis for associations with dichotomized mRS at 3 months Outcome was measured as mRS 0–2 vs 3–6 at 3 months after ischemic stroke onset. Dotted lines show genome-wide significance (black, *p* <5 × 10^−8^) and suggestive association level (green, *p* < 10^−5^). Results are adjusted for age, sex, principal components, and baseline NIH Stroke Scale score. mRS = modified Rankin Scale.

**Figure 2:**
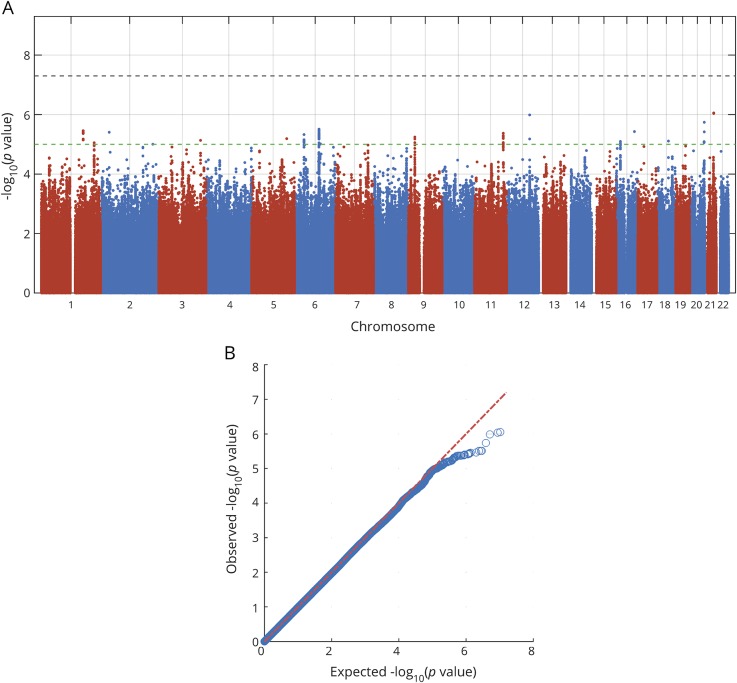
Manhattan and quantile-quantile plots of analysis for associations with ordinal mRS at 3 months Outcome was measured as ordinal mRS at 3 months after ischemic stroke onset. Dotted lines show genome-wide significance (black, *p* <5 × 10^−8^) and suggestive association level (green, *p* < 10^−5^). Results are adjusted for age, sex, principal components, and baseline NIH Stroke Scale score. mRS = modified Rankin Scale.

Inverse variance-weighted fixed-effects meta-analysis was performed using METAL software.^12^ Variants with minor allele frequency <0.01 were excluded. Variants that were missing in >50% of cohorts were also excluded. After filtering, approximately 8.5 million SNPs were included in each of the final meta-analyses. Quantile-quantile plots are shown in figures 1 and 2 and in data available from Dryad (figures e-1 to e-4, doi.org/10.5061/dryad.s38kf65), and genomic inflation factors (λ) are displayed in data available from Dryad (table e-4). There was no sign of population stratification based on these measures. Heterogeneity of SNP effects between studies was tested in the meta-analysis.

Markers with *p* values <5 × 10^−8^ were considered significant for association with outcome, while markers with *p* values <1 × 10^−5^ were considered suggestive. To facilitate comparison of the results from the dichotomized and ordinal analyses, we present all effect sizes as odds ratios (ORs) per copy of the minor allele; an OR >1 indicates a higher mRS score (worse outcome) per copy of the minor allele and an OR <1 indicates a lower mRS score.

### Investigation of expression quantitative trait loci

We explored associations of the markers with *p* values <1 × 10^−5^ and proxy SNPs (*r*^2^ > 0.8 in 1000 Genomes Phase 1 European population) with expression quantitative trait loci (eQTLs) in publicly available datasets encompassing numerous tissues: Genotype-Tissue Expression (GTEx) V6,^13^ GRASP2,^14,15^ Human Genetic Variation Database (HGVD),^16^ and BIOS.^17^ For eQTLs, *p* < 10^−4^ was considered significant.

### Gene-based analysis

Gene-based tests were performed for each meta-analysis using VEGAS2 with linkage disequilibrium structure based on the European population.^18^ All SNPs within ±10 kbp from the untranslated regions 3′ and 5′ of each gene were included, to account for potential regulatory variants.^18^ The number of genes included was approximately 23,000, which corresponds to a Bonferroni-corrected significance threshold of *p* < 2.2 × 10^−6^.

### Data availability

The datasets generated and analyzed during the current study are available on reasonable request.

## Results

Characteristics such as age, sex, and stroke severity, as well as the numbers of included cases for each mRS score and in each outcome analysis, are displayed in table 1. The analyses of mRS 0–2 vs 3–6 and the ordinal analyses included 6,021 stroke cases, whereas analyses of mRS 0–1 vs 2–6 included 4,363 cases.

**Table 1:**
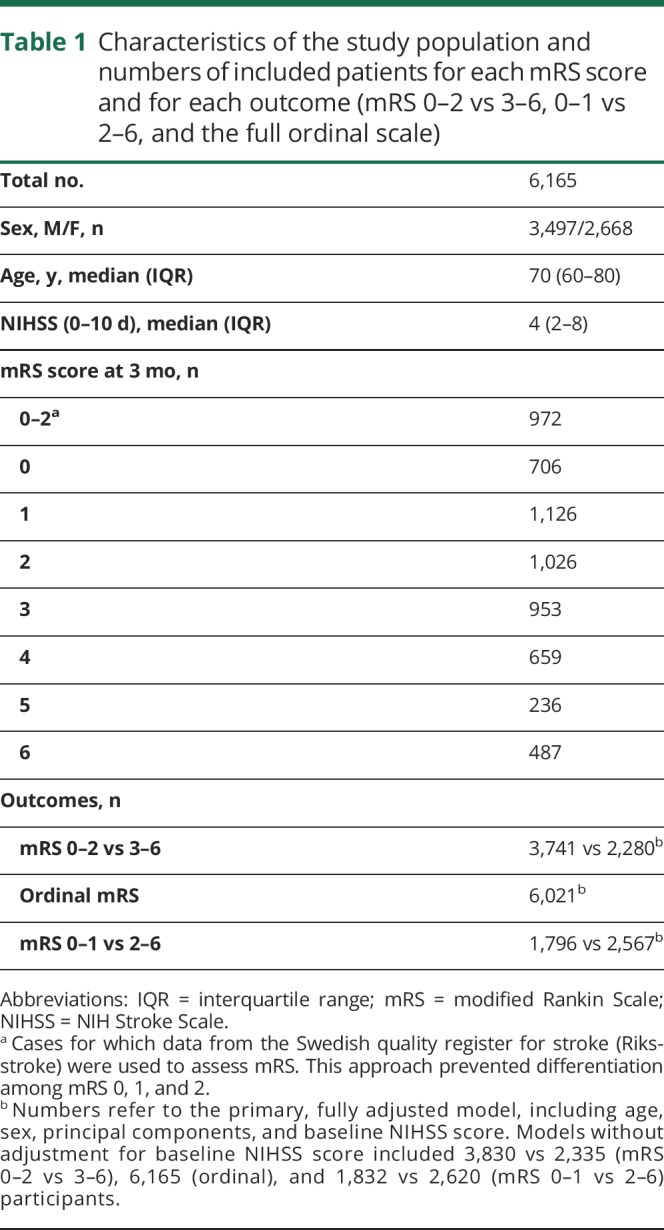
Characteristics of the study population and numbers of included patients for each mRS score and for each outcome (mRS 0–2 vs 3–6, 0–1 vs 2–6, and the full ordinal scale)

**Figure 3:**
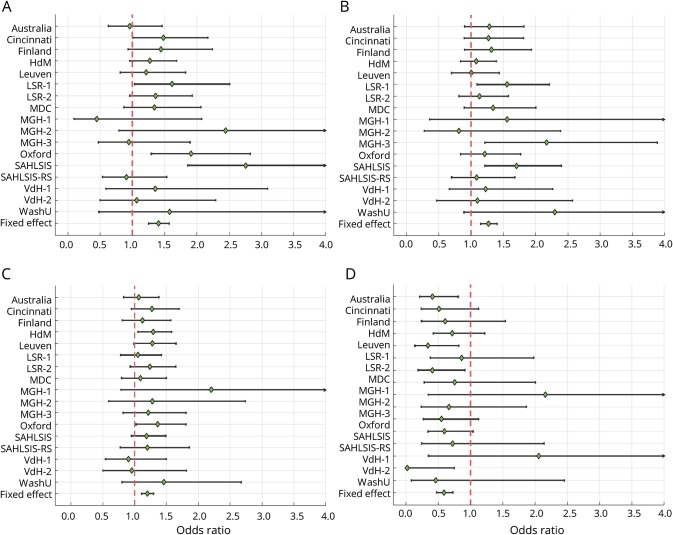
Forest plots for functional outcome at 3 months by study cohort The plots show ORs and 95% confidence intervals for minor allele of (A) rs1842681 (mRS 0–2 vs 3–6), (B) rs2236406 (mRS 0–2 vs 3–6), (C) rs13299556 (ordinal mRS), and (D) rs78734480 (ordinal mRS). ORs with effects above unity indicate a higher mRS score (worse outcome) per copy of the minor allele. Results are adjusted for age, sex, principal components, and baseline NIH Stroke Scale score. For cohort abbreviations, see data available from Dryad (table e-1, doi.org/10.5061/dryad.s38kf65). mRS = modified Rankin Scale; OR = odds ratio.

**Figure 4:**
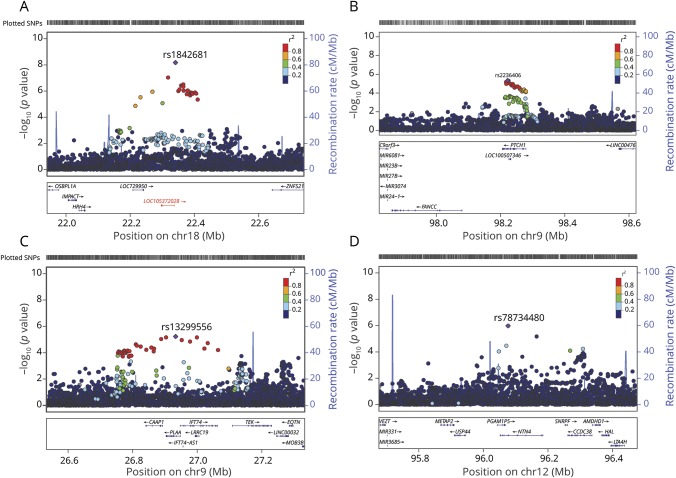
Regional association plots for outcome at 3 months after ischemic stroke onset (A) Significant locus (rs1842681) showing association with mRS 0–2 vs 3–6, (B) rs2236406 (intron variant in the *PTCH1* gene) showing suggestive association with mRS 0–2 vs 3–6, (C) rs13299556 (intron variant in the *PLAA* gene with eQTL for *TEK*, *p*_*GTEx*_ = 1.6 × 10^−6^) showing suggestive association with ordinal mRS, and (D) rs78734480 (intron variant in the *NTN4* gene) showing suggestive association with ordinal mRS. Results are adjusted for age, sex, principal components, and baseline NIH Stroke Scale score. *LOC105372028* (indicated in red, panel A) has been overlaid from the Genome Reference Consortium Human Build (GRCh) 38/hg38, as it was missing from GRCh37/hg19. The rs1842681 variant is intronic of the *LOC105372028* gene (chromosome 18: 24725781–24766645). Position for rs1842681 in GRCh38/hg38, 18:24761199. eQTL = expression quantitative trait locus; GTEx = Genotype-Tissue Expression; mRS = modified Rankin Scale; SNP = single-nucleotide polymorphism.

One common variant on chromosome 18q11.2 (rs1842681, minor allele frequency: 0.23) was associated at genome-wide significance with outcome defined as mRS 0–2 vs 3–6 (OR for minor allele [A]: 1.40, *p* = 5.3 × 10^−9^) (table 2, figures 1, 3, and 4). The effect was similar with and without adjustment for stroke severity (table 2), and the association was observed in the same direction, but with a somewhat lower effect size, for ordinal mRS (OR: 1.17, *p* = 1.5 × 10^−4^) and mRS 0–1 vs 2–6 (OR: 1.12, *p* = 7.4 × 10^−2^). In line with this, the distribution of the minor allele count for rs1842681 over mRS categories shows a threshold between mRS 2 and 3 (data available from Dryad, figure e-5, doi.org/10.5061/dryad.s38kf65). The variant is located in an intron of the gene *LOC105372028* (long noncoding RNA synonymous with *RP11-449D8.5* [Genome Reference Consortium Human Build 38/hg38]) and is in a putative binding site of several regulatory proteins (HaploReg, March 21, 2018). This variant has no eQTL reported in GTEx (June 1, 2018). However, in HGVD, it has a trans-eQTL for *KLRAQ1* (also known as *PPP1R21*, *p*_*HGVD*_ = 1.67 ×10^−7^). *PPP1R21* is expressed in the brain (GTEx, June 1, 2018).

**Table 2:**
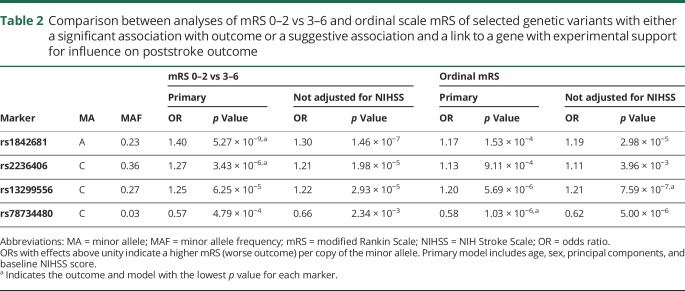
Comparison between analyses of mRS 0–2 vs 3–6 and ordinal scale mRS of selected genetic variants with either a significant association with outcome or a suggestive association and a link to a gene with experimental support for influence on poststroke outcome

No other SNP was significantly associated with mRS 0–2 vs 3–6, or with ordinal scale mRS (figures 1 and 2). The results for the lead SNPs of the top 10 independent loci for each outcome are displayed in data available from Dryad (tables e-5 and e-6, doi.org/10.5061/dryad.s38kf65). The results from analyses without severity adjustment are displayed in data available from Dryad (tables e-7 and e-8 and figures e-1, e-2, e-6, and e-7). There were no signs of heterogeneity for the effects of the reported SNPs. All *I*^2^ values were <0.35 and all *p* for heterogeneity >0.08, except for one SNP (rs58448576) in the analysis of mRS 0–1 vs 2–6, which had an *I*^2^ of 0.48 and a *p* value of 0.03 (without adjustment for multiple testing). In the gene-based analyses, no gene reached the predefined threshold for significance (data available from Dryad, table e-9).

The analysis of mRS 0–1 vs 2–6 yielded no significant associations. These results are presented in data available from Dryad (tables e-12 and e-13 and figures e-3, e-4, and e-8, doi.org/10.5061/dryad.s38kf65).

### Suggestive associations with outcome

Thirty-three SNPs in 12 different loci (with at least 1 Mbp distance) were suggestively (*p* < 10^−5^) associated with mRS 0–2 vs 3–6 (excluding the SNPs in the significant locus on chromosome 18q11.2), and 75 SNPs in 17 different loci with ordinal mRS (figures 1 and 2). Of these 29 independent loci, the top SNPs of 16 loci have either significant eQTLs for and/or are located within or near (<100 kbp) genes that are expressed in the brain (GTEx, June 1, 2018). Five genes that were not reported to be expressed in brain tissue were expressed in arteries or lymphocytes (GTEx, June 1, 2018).

Among the suggestive associations, 3 are linked to genes with experimental evidence of influence on outcome from animal models of stroke and are discussed below.^19–21^ First, rs2236406, an intron variant in the *PTCH1* gene, was identified in the mRS 0–2 vs 3–6 analysis (table 2, figures 3) and 4). The gene-based analysis also showed a suggestive association for *PTCH1* with mRS 0–2 vs 3–6 (*p* = 6.8 × 10^−5^). Rs2236406 is an eQTL for long noncoding RNA RP11-435O5.5 (*p*_*GTEx*_ = 5.7 × 10^−7^), which overlaps the *PTCH1* gene. Second, in the ordinal analysis, a suggestive association with mRS was found for rs13299556 (table 2, figures 3) and 4). Associations at low *p* values were also found for this variant with dichotomous mRS, although not reaching the level for suggestive association (table 2). rs13299556 is an intron variant in the *PLAA* gene. There was no strong association for *PLAA* in the gene-based analysis (ordinal mRS; *p* = 0.072). However, this variant, and variants in high linkage disequilibrium with it (figure 4), are reported as eQTLs for the nearby genes *TEK* and *LRRC19* (*p*_*GTEx*_ = 1.6 × 10^−6^ for both). The *p* values from the gene-based analysis for those genes were 0.0019 and 0.084, respectively. The variant is also predicted to alter a putative regulatory motif sequence (HaploReg, March 21, 2018). Third, a suggestive association with ordinal mRS was found for an intron variant in *NTN4* (rs78734480; table 2, figures 3 and 4). This variant was also associated with dichotomous mRS at a low *p* value, although not below the cutoff for suggestive association (table 2).

### Analysis of lacunar and nonlacunar stroke

To evaluate whether any strong associations could be identified specifically in patients with lacunar stroke (small vessel disease strokes, n = 992), or in nonlacunar stroke (other etiologic subtypes including cortical infarcts, n = 3,991), we analyzed those groups separately. No genome-wide significant association was detected (all *p* values >6 × 10^−7^). The top 5 findings differed between these subgroups and are shown in data available from Dryad (tables e-10 and e-11, doi.org/10.5061/dryad.s38kf65).

## Discussion

This GWA study on functional outcome after ischemic stroke, including more than 6,000 patients, identified one significant locus and several suggestive variants related to genes with a potential mechanism for influencing stroke recovery and outcomes. Replication of these findings in independent datasets as they become available is the essential next step. The effect sizes of these genetic variants were generally small, and further studies should include even larger samples to identify additional variants associated with stroke outcome and to enable subgroup analyses.

The genome-wide significant SNP (rs1842681) for mRS 0–2 vs 3–6 is an intronic variant in the gene *LOC105372028* (long noncoding RNA), the function of which remains to be established. However, expression analyses show that the gene expression of *LOC105372028* is highest in brain tissue (National Center for Biotechnology Information, ncbi.nlm.nih.gov/gene/?term=LOC105372028) and rs1842681 is located in a putative regulatory element binding site (HaploReg, March 21, 2018). Furthermore, rs1842681 is a trans-eQTL for *KLRAQ1*, also known as *PPP1R21*, which encodes a regulatory subunit of protein phosphatase 1 (PP1). PP1 is a ubiquitous phosphatase implicated in many brain functions including learning and memory formation.^22,23^ PP1 is also a key regulator of Ca^2+^/calmodulin (CaM)-dependent protein kinase II (CaMKII) signaling, which is crucial for Ca^2+^-mediated neuronal plasticity in the brain.^24^ Thus, although speculative, rs1842681 may modulate expression of PPP1R21, which in turn could affect brain plasticity and thereby outcome post stroke, a hypothesis that requires validation through functional experiments. The association for rs1842681 with outcome was observed after adjusting for baseline NIHSS score, suggesting a mechanism that is independent of initial stroke severity. However, additional data from further studies are clearly needed to corroborate this finding and clarify the potential role of this locus for functional outcome after stroke.

Twenty-nine independent loci were associated with ischemic stroke outcome at the predefined suggestive association level. Three of these loci are linked to genes with experimental evidence of influence on outcome from animal models of stroke.^19–21^ First, the intronic variant in *PLAA* (rs13299556) was both one of the top findings in the ordinal analysis and associated with the dichotomized outcome at a low *p* value. It is located in a putative transcription factor binding site and is reported to be an eQTL for the expression of both *LRRC19* and *TEK*. *LRRC19* encodes a protein with a potential role in regulating neurite outgrowth,^25^ and might thus influence stroke recovery. *TEK* encodes a tyrosine kinase predominantly expressed in endothelial cells (ECs). Focal upregulation of *TEK* has been demonstrated in capillaries at the border of infarcted myocardium, and expression is induced by hypoxia and proinflammatory cytokines in human ECs in vitro*.*^26^ *TEK* is activated by angiopoietin-1 and this promotes migration, sprouting, and survival of ECs.^27,28^ Of note, results from a rodent ischemic stroke model suggest that upregulation of angiopoietin-1 and *TEK* improves stroke outcome,^19^ and one clinical study reported an association between high plasma levels of angiopoietin-1 and poor outcome as assessed by mRS at 3 months after ischemic stroke.^29^ Angiopoietin-2 is the antagonist of angiopoietin-1.^30^ Angiopoietin-2 gain-of-function mice have enhanced blood-brain barrier permeability and increased brain infarct sizes upon permanent middle cerebral occlusion compared to wild-type mice, and both phenotypes were rescued by activation of *TEK* signaling.^31^ The same study reported increased circulating serum concentrations of angiopoietin-2 in patients with acute ischemic stroke compared to controls.^31^ Finally, in an ischemic stroke model, mice with type 2 diabetes mellitus showed increased angiopoietin-2 but decreased angiopoietin-1/TEK protein expression compared to wild-type mice, suggesting that the TEK signaling may be involved in diabetes-induced vascular damage post stroke.^32^

Second, an intronic variant in the *PTCH1* gene showed suggestive association with mRS 0–2 vs 3–6. *PTCH1* is involved in sonic hedgehog signaling, a pathway that for instance reduces oxidative stress on neurons and regulates ischemia-induced neuronal progenitor proliferation.^33,34^ In rats with experimentally induced middle cerebral artery occlusion, inhibition of the sonic hedgehog signaling caused increased infarct size, and *PTCH1* was downregulated when sonic hedgehog signaling was inhibited.^20,35^ The association between *PTCH1* and stroke outcome observed in the present study may thus potentially be explained by an increased infarct size. However, the effect of this variant was somewhat stronger when adjusting for baseline NIHSS score, which may suggest a potential influence also on recovery processes.

Third, a variant in *NTN4* showed suggestive association with ordinal mRS and was also associated with dichotomous mRS at a low *p* value. *NTN4* encodes a member of the netrin family of proteins expressed in brain tissue (ncbi.nlm.nih.gov/gene/59277) and with functions in processes with biologically plausible roles in stroke recovery such as angiogenesis, neurite growth, and migration.^36,37^ In a mouse model, *NTN4* was upregulated in blood vessels and astrocytes in the ischemic core after stroke, and intracerebroventricular administration of NTN4 led to improvements both in angiogenesis and in poststroke outcome, possibly through increased collateral blood flow leading to improved survival of neurons partially affected by ischemia.^21^ In another study, *NTN4* contributed to thalamocortical axon branching in rodents and the expression was altered by neuronal activity in the cortex, implying that *NTN4* might act as a positive regulator for thalamocortical axon branching through activity-dependent expression.^38^ Although highly speculative, this gene could thus have a role in the plastic process of axonal outgrowth and restoration of synaptic architecture post stroke.

Several additional variants with suggestive association to outcome in this study are linked to genes that may potentially be mechanistically involved in processes affecting stroke outcomes. For instance, *RUNX1* encodes a runt-related proangiogenic transcription factor with high expression in arteries (GTEx, June 1, 2018) that is upregulated after stroke.^39^ In mice, expression of *RUNX1* is induced in putative neural stem or progenitor cells after brain injury and suggested to promote neuronal differentiation.^40^ *TNR* encodes a protein that is involved in neuronal plasticity and is highly and exclusively expressed in the brain.^41,42^ Examples of genes with a possible stroke subtype-specific significance among suggestive findings are *MTHFS*, which is involved in folate and homocysteine metabolism, pathways with potential influence on cerebral small vessel disease,^43^ and *SCML4*, in which the variant rs74514008 was suggestively associated with ordinal mRS in the present study. *SCML4* has shown association with coronary artery disease in a recent GWA study, and subsequent functional characterization suggested a role in atherosclerosis.^44^

To further explore the SNPs associated with outcomes in different subgroups, such as certain stroke subtypes, patients with diabetes, or those receiving recanalization therapies would be of great interest. Mechanisms of neuron injury and recovery probably have similarities regardless of the etiology, but mechanism of recovery after cortical and subcortical stroke may be different. Therefore, we analyzed mRS 0–2 vs 3–6 in patients with lacunar and nonlacunar stroke separately. The top 5 independent loci differed between the 2 groups, but no genome-wide significant association was detected. Given the small sample sizes, especially for lacunar stroke, these analyses are clearly exploratory. Thus, in further studies with a higher number of patients, an important aim will be to further explore the genetic associations in specific subgroups.

The strengths of the present study include a relatively large sample size, well defined endpoints, extensive genetic data, and that analyses were adjusted for NIHSS score to assess the influence on outcome independent of baseline stroke severity. We performed dichotomous outcome analyses to identify genetic variants associated with being dependent or independent in activities of daily living, which reflects a clinically important difference in functional outcome. We also performed ordinal analysis, which aims to identify variants that have a similar effect across different degrees of functional outcome. To detect such variants, the ordinal model provides greater power.^8^ Another strength of the ordinal approach is the ability to compensate for any differences in mRS assessment between studies.

There are also several limitations that should be considered. Although this study included more than 6,000 patients with ischemic stroke, only one significant locus was identified, and thus, the findings do not explain the assumed genetic variation for ischemic stroke outcomes. This implies that the effect sizes of individual SNPs on outcome of ischemic stroke are small and that our sample size might be insufficient even to detect common genetic variants. Another important limitation is the explorative nature of the results, which clearly require future replication in independent cohorts as they become available. The use of the mRS as the primary outcome metrics is a clear limitation in that it is a crude measurement of outcome, especially when dichotomized. However, the mRS represents a well-established, formalized, and easily available measure of poststroke functional outcome, and the dichotomizations represent differences of great importance clinically and for the patient. The mRS scores were assessed at different time points ranging from 60 to 190 days, potentially diluting any detectable associations, as functional outcome status may vary over time. However, the majority of included studies assessed mRS at 3 months ± 2 weeks after stroke onset. We did not have information on premorbid mRS and this would have provided a valuable means to calculate the change in functional ability. Moreover, stroke severity was scored at different time points. However, in a majority of cases, NIHSS was scored early and in only 160 individuals later than day 3 after admission. As previously described,^8^ we lack data on some important factors that influence outcomes such as acute therapies and rehabilitation, which is why these factors could not be accounted for in the analyses. However, data from the cohorts with available data on treatment with tissue plasminogen activator (about half of the cohorts) show that this treatment was given to 14% of the patients. We can also not exclude the possibility of selection bias since participation in the study required individual informed consent, and data entry also requested availability of functional status follow-up data. In line with this, our study sample mainly reflects milder strokes (median NIHSS score of 4, table 1), which may hamper the detection of factors influencing a greater dynamic range of recovery. Lastly, because our study population was of European ancestry, the results are not necessarily generalizable to other stroke populations. Further studies should ideally include a series of prespecified collection time points, specific clinical variables of importance for stroke outcome, and a battery of outcome metrics that assess several domains of recovery including motor impairment, aphasia, neglect, and cognitive impairment.

In this first large international GWA study on functional outcome after overall ischemic stroke, we report one significant variant and several variants with suggestive association to outcome at 3 months after stroke onset with plausible links to poststroke recovery. Future studies on common variants should include larger sample sizes, enabling subgroup analyses, as well as replication of the present results and elaboration of potential mechanisms.

## Author Affiliation

From the Department of Clinical Sciences Malmö (M.S.) and Department of Clinical Sciences Lund, Neurology (B.N., A.L.), Lund University; Department of Neurology and Rehabilitation Medicine (M.S., B.N., A.L.), Neurology, Skåne University Hospital, Lund and Malmö; Institute of Biomedicine (A.P., T.M.S., M.O., C.J.) and Department of Clinical Neuroscience Institute of Neuroscience and Physiology (K.J., T.T.), Sahlgrenska Academy at the University of Gothenburg; Department of Clinical Genetics and Genomics (A.P., C.J.) and Department of Neurology (K.J.), Sahlgrenska University Hospital, Gothenburg; Bioinformatics Core Facility (E.L.), University of Gothenburg, Sweden; School of Life Sciences (S.B.), University of Lincoln, UK; Department of Neurology (J.C.), University of Maryland School of Medicine and Baltimore VAMC; Stroke Pharmacogenomics and Genetics (I.F.-C.), Institut d'investigació Biomedica de Sant Pau, Hospital de Sant Pau; Neurovascular Research Laboratory and Neurovascular Unit (I.F.-C.), Neurology and Medicine Departments, Universitat Autònoma de Barcelona, Vall d'Hebrón Hospital, Barcelona, Spain; Medical School (G.J.H.), The University of Western Australia, Perth; Department of Neurology (J.J.-C.), Institut Hospital del Mar d'Investigació Mèdica (IMIM), Barcelona; Universitat Autònoma de Barcelona (J.J.-C.), Barcelona, Spain; Department of Neurology (J.-M.L.), Washington University School of Medicine, St. Louis, MO; Department of Neurosciences (R.L.), Experimental Neurology, KU Leuven, University of Leuven; Center for Brain & Disease Research (R.L.), Laboratory of Neurobiology, VIB, Leuven; Department of Neurology (R.L.), University Hospitals Leuven, Belgium; School of Medicine and Public Health (C.L., R.S., J.S.), Hunter Medical Research Institute (C.L., R.S.), and Priority Research Centre for Stroke and Traumatic Brain Injury (C.L.), University of Newcastle, Australia; Department of Medicine (B.D.M.), University of Maryland, Baltimore; Geriatric Research, Education, and Clinical Center (B.D.M.), Veterans Affairs Medical Center, Baltimore, MD; Centre for Clinical Brain Sciences (K.R., C.S.), University of Edinburgh, UK; J. Philip Kistler Stroke Research Center, Department of Neurology (N.R., J.R.), Massachusetts General Hospital, Harvard Medical School, Boston; Program in Medical and Population Genetics (J.R.), Broad Institute of MIT and Harvard, Cambridge; Center for Genomic Medicine (J.R.), Massachusetts General Hospital, Boston; Stroke Prevention Research Unit (P.M.R.), Nuffield Department of Clinical Neurosciences, University of Oxford, UK; Department of Neurology (D.S., T.T.), Helsinki University Hospital, Finland; Department of Clinical Neurosciences (M.T.), University of Cambridge, UK; Stroke Division (V.T.), Florey Institute for Neuroscience and Mental Health, University of Melbourne, Heidelberg; Department of Neurology (V.T.), Austin Health, Heidelberg, Australia; Department of Neurology and Rehabilitation (D.W.), University of Cincinnati College of Medicine, OH; Departments of Neurology and Health Evaluation Sciences (B.B.W.), University of Virginia, Charlottesville; University of Technology Sydney (J.M.M.), Faculty of Health, Australia; and Hunter Medical Research Centre (J.M.M.), PRC Stroke and Brain Injury, Newcastle, Australia.

## Glossary

EC: endothelial cell
eQTL: expression quantitative trait locus
GISCOME: Genetics of Ischaemic Stroke Functional Outcome
GTEx: Genotype-Tissue Expression
GWA: genome-wide association
HGVD: Human Genetic Variation Database
mRS: modified Rankin Scale
NIHSS: NIH Stroke Scale
OR: odds ratio
PP1: protein phosphatase 1
SNP: single-nucleotide polymorphism

## References

[1] Lindgren A, Maguire J. Stroke recovery genetics. Stroke 2016;47:2427–2434. 27515845 10.1161/STROKEAHA.116.010648

[2] McAllister TW. Genetic factors modulating outcome after neurotrauma. PM R 2010;2:S241–S252. 21172686 10.1016/j.pmrj.2010.10.005

[3] Pearson-Fuhrhop KM, Burke E, Cramer SC. The influence of genetic factors on brain plasticity and recovery after neural injury. Curr Opin Neurol 2012;25:682–688. 23044515 10.1097/WCO.0b013e32835a360a

[4] Goldberg A, Curtis CL, Kleim JA. Linking genes to neurological clinical practice: the genomic basis for neurorehabilitation. J Neurol Phys Ther 2015;39:52–61. 25415554 10.1097/NPT.0000000000000066

[5] Zhao J, Wu H, Zheng L, Weng Y, Mo Y. Brain-derived neurotrophic factor G196A polymorphism predicts 90-day outcome of ischemic stroke in Chinese: a novel finding. Brain Res 2013;1537:312–318. 24035862 10.1016/j.brainres.2013.08.061

[6] Stanne TM, Tjärnlund-Wolf A, Olsson S, Jood K, Blomstrand C, Jern C. Genetic variation at the BDNF locus: evidence for association with long-term outcome after ischemic stroke. PLoS One 2014;9:e114156. 25470006 10.1371/journal.pone.0114156 PMC4254920

[7] Maguire J, Thakkinstian A, Levi C, et al. Impact of COX-2 rs5275 and rs20417 and GPIIIa rs5918 polymorphisms on 90-day ischemic stroke functional outcome: a novel finding. J Stroke Cerebrovasc Dis 2011;20:134–144. 20472470 10.1016/j.jstrokecerebrovasdis.2009.10.011

[8] Maguire J, Bevan S, Stanne TM, et al. GISCOME—Genetics of Ischaemic Stroke Functional Outcome network: a protocol for an international multicentre genetic association study. Eur Stroke J 2017;2:229–237. 31008316 10.1177/2396987317704547 PMC6454830

[9] Eriksson M, Appelros P, Norrving B, Terent A, Stegmayr B. Assessment of functional outcome in a national quality register for acute stroke: can simple self-reported items be transformed into the modified Rankin Scale? Stroke 2007;38:1384–1386. 17322093 10.1161/01.STR.0000260102.97954.9c

[10] Adams HP Jr, Bendixen BH, Kappelle LJ, et al. Classification of subtype of acute ischemic stroke. Definitions for use in a multicenter clinical trial. TOAST. Trial of Org 10172 in Acute Stroke Treatment. Stroke 1993;24:35–41. 7678184 10.1161/01.str.24.1.35

[11] Chang CC, Chow CC, Tellier LC, Vattikuti S, Purcell SM, Lee JJ. Second-generation PLINK: rising to the challenge of larger and richer datasets. Gigascience 2015;4:7. 25722852 10.1186/s13742-015-0047-8 PMC4342193

[12] Willer CJ, Li Y, Abecasis GR. METAL: fast and efficient meta-analysis of genomewide association scans. Bioinformatics 2010;26:2190–2191. 20616382 10.1093/bioinformatics/btq340 PMC2922887

[13] GTEx Consortium. Human genomics. The Genotype-Tissue Expression (GTEx) pilot analysis: multitissue gene regulation in humans. Science 2015;348:648–660. 25954001 10.1126/science.1262110 PMC4547484

[14] Eicher JD, Landowski C, Stackhouse B, et al. GRASP v2.0: an update on the Genome-Wide Repository of Associations between SNPs and phenotypes. Nucleic Acids Res 2015;43:D799–D804. 25428361 10.1093/nar/gku1202 PMC4383982

[15] Leslie R, O'Donnell CJ, Johnson AD. GRASP: analysis of genotype-phenotype results from 1,390 genome-wide association studies and corresponding open access database. Bioinformatics 2014;30:i185–i194. 24931982 10.1093/bioinformatics/btu273 PMC4072913

[16] Higasa K, Miyake N, Yoshimura J, et al. Human genetic variation database, a reference database of genetic variations in the Japanese population. J Hum Genet 2016;61:547–553. 26911352 10.1038/jhg.2016.12 PMC4931044

[17] Bonder MJ, Luijk R, Zhernakova DV, et al. Disease variants alter transcription factor levels and methylation of their binding sites. Nat Genet 2017;49:131–138. 27918535 10.1038/ng.3721

[18] Mishra A, Macgregor S. VEGAS2: software for more flexible gene-based testing. Twin Res Hum Genet 2015;18:86–91. 25518859 10.1017/thg.2014.79

[19] Zheng Q, Zhu D, Bai Y, Wu Y, Jia J, Hu Y. Exercise improves recovery after ischemic brain injury by inducing the expression of angiopoietin-1 and Tie-2 in rats. Tohoku J Exp Med 2011;224:221–228. 21701128 10.1620/tjem.224.221

[20] Ji H, Miao J, Zhang X, et al. Inhibition of sonic hedgehog signaling aggravates brain damage associated with the down-regulation of Gli1, Ptch1 and SOD1 expression in acute ischemic stroke. Neurosci Lett 2012;506:1–6. 22133807 10.1016/j.neulet.2011.11.027

[21] Hoang S, Liauw J, Choi M, Choi M, Guzman RG, Steinberg GK. Netrin-4 enhances angiogenesis and neurologic outcome after cerebral ischemia. J Cereb Blood Flow Metab 2009;29:385–397. 18985053 10.1038/jcbfm.2008.128

[22] Giese KP, Mizuno K. The roles of protein kinases in learning and memory. Learn Mem 2013;20:540–552. 24042850 10.1101/lm.028449.112

[23] Heroes E, Lesage B, Gornemann J, Beullens M, Van Meervelt L, Bollen M. The PP1 binding code: a molecular-lego strategy that governs specificity. FEBS J 2013;280:584–595. 22360570 10.1111/j.1742-4658.2012.08547.x

[24] Shioda N, Fukunaga K. Physiological and pathological roles of CaMKII-PP1 signaling in the brain. Int J Mol Sci 2017;19:E20. 29271887 10.3390/ijms19010020 PMC5795971

[25] Aruga J, Mikoshiba K. Identification and characterization of Slitrk, a novel neuronal transmembrane protein family controlling neurite outgrowth. Mol Cell Neurosci 2003;24:117–129. 14550773 10.1016/s1044-7431(03)00129-5

[26] Willam C, Koehne P, Jurgensen JS, et al. Tie2 receptor expression is stimulated by hypoxia and proinflammatory cytokines in human endothelial cells. Circ Res 2000;87:370–377. 10969034 10.1161/01.res.87.5.370

[27] Suri C, Jones PF, Patan S, et al. Requisite role of angiopoietin-1, a ligand for the TIE2 receptor, during embryonic angiogenesis. Cell 1996;87:1171–1180. 8980224 10.1016/s0092-8674(00)81813-9

[28] Suri C, McClain J, Thurston G, et al. Increased vascularization in mice overexpressing angiopoietin-1. Science 1998;282:468–471. 9774272 10.1126/science.282.5388.468

[29] Golledge J, Clancy P, Maguire J, et al. Plasma angiopoietin-1 is lower after ischemic stroke and associated with major disability but not stroke incidence. Stroke 2014;45:1064–1068. 24569814 10.1161/STROKEAHA.113.004339

[30] Maisonpierre PC, Suri C, Jones PF, et al. Angiopoietin-2, a natural antagonist for Tie2 that disrupts in vivo angiogenesis. Science 1997;277:55–60. 9204896 10.1126/science.277.5322.55

[31] Gurnik S, Devraj K, Macas J, et al. Angiopoietin-2-induced blood-brain barrier compromise and increased stroke size are rescued by VE-PTP-dependent restoration of Tie2 signaling. Acta Neuropathol 2016;131:753–773. 26932603 10.1007/s00401-016-1551-3 PMC4835530

[32] Cui X, Chopp M, Zacharek A, Ye X, Roberts C, Chen J. Angiopoietin/Tie2 pathway mediates type 2 diabetes induced vascular damage after cerebral stroke. Neurobiol Dis 2011;43:285–292. 21515377 10.1016/j.nbd.2011.04.005 PMC3096677

[33] Dai RL, Zhu SY, Xia YP, et al. Sonic hedgehog protects cortical neurons against oxidative stress. Neurochem Res 2011;36:67–75. 20848190 10.1007/s11064-010-0264-6

[34] Sims JR, Lee SW, Topalkara K, et al. Sonic hedgehog regulates ischemia/hypoxia-induced neural progenitor proliferation. Stroke 2009;40:3618–3626. 19762700 10.1161/STROKEAHA.109.561951 PMC2869495

[35] Ji H, Zhang X, Du Y, Liu H, Li S, Li L. Polydatin modulates inflammation by decreasing NF-kappaB activation and oxidative stress by increasing Gli1, Ptch1, SOD1 expression and ameliorates blood-brain barrier permeability for its neuroprotective effect in pMCAO rat brain. Brain Res Bull 2012;87:50–59. 22001340 10.1016/j.brainresbull.2011.09.021

[36] Koch M, Murrell JR, Hunter DD, et al. A novel member of the netrin family, beta-netrin, shares homology with the beta chain of laminin: identification, expression, and functional characterization. J Cell Biol 2000;151:221–234. 11038171 10.1083/jcb.151.2.221 PMC2192657

[37] Zhang C, Meng F, Wang C, et al. Identification of a novel alternative splicing form of human netrin-4 and analyzing the expression patterns in adult rat brain. Brain Res Mol Brain Res 2004;130:68–80. 15519678 10.1016/j.molbrainres.2004.07.009

[38] Hayano Y, Sasaki K, Ohmura N, et al. Netrin-4 regulates thalamocortical axon branching in an activity-dependent fashion. Proc Natl Acad Sci USA 2014;111:15226–15231. 25288737 10.1073/pnas.1402095111 PMC4210296

[39] Buga AM, Margaritescu C, Scholz CJ, Radu E, Zelenak C, Popa-Wagner A. Transcriptomics of post-stroke angiogenesis in the aged brain. Front Aging Neurosci 2014;6:44. 24672479 10.3389/fnagi.2014.00044 PMC3957426

[40] Logan TT, Rusnak M, Symes AJ. Runx1 promotes proliferation and neuronal differentiation in adult mouse neurosphere cultures. Stem Cell Res 2015;15:554–564. 26473321 10.1016/j.scr.2015.09.014

[41] Hargus G, Cui Y, Schmid JS, et al. Tenascin-R promotes neuronal differentiation of embryonic stem cells and recruitment of host-derived neural precursor cells after excitotoxic lesion of the mouse striatum. Stem Cell 2008;26:1973–1984. 10.1634/stemcells.2007-0929 18499893

[42] Xu JC, Xiao MF, Jakovcevski I, et al. The extracellular matrix glycoprotein tenascin-R regulates neurogenesis during development and in the adult dentate gyrus of mice. J Cell Sci 2014;127:641–652. 24338367 10.1242/jcs.137612

[43] Rutten-Jacobs LC, Traylor M, Adib-Samii P, et al. Association of MTHFR C677T genotype with ischemic stroke is confined to cerebral small vessel disease subtype. Stroke 2016;47:646–651. 26839351 10.1161/STROKEAHA.115.011545 PMC4760380

[44] Li Y, Wang DW, Chen Y, et al. Genome-wide association and functional studies identify SCML4 and THSD7A as novel susceptibility genes for coronary artery disease. Arterioscler Thromb Vasc Biol 2018;38:964–975. 29472232 10.1161/ATVBAHA.117.310594

